# Giant Lipoma of the Bauhin’s Valve

**DOI:** 10.3390/diagnostics14050562

**Published:** 2024-03-06

**Authors:** Cosmina Fugărețu, Catalin Misarca

**Affiliations:** 1 2nd General Surgery Department, Brașov County Emergency Clinical Hospital, 500326 Brașov, Romania; cmisarca@gmail.com; 2 Faculty of General Medicine Brașov, Transilvania University, 500019 Brașov, Romania

**Keywords:** intestinal lipoma, lipoma of the ileocecal valve, lower gastrointestinal bleeding

## Abstract

Lipomas are benign tumors that can affect the digestive tract, everywhere from the hypopharynx to the rectum. Lipomas affecting the large intestine are the second most common benign tumor, after colon adenoma. We present the case of a 46-year-old patient who was initially hospitalized in the Gastroenterology Clinic with a diagnosis of gastrointestinal bleeding. The colonoscopy raised the suspicion of a malignant tumor of the transverse colon, but the computed tomography scan showed the existence of a lipoma that measured 16/11/12 cm that occupied the ascending and transverse colon, though the CT examination could not determinate the origin of the lipoma. After restoring the hydro-electrolytic and fluid balance of the patient, surgery was performed and a huge lipoma of the ileocecal valve was discovered. Extended right hemicolectomy was performed, with good subsequent postoperative recovery of the patient, who was discharged on the fifth day after the surgery. The peculiarity of this case is the huge size of the benign tumor. Lipomas with digestive system localization, although rare, must be considered in patients arriving at the Emergency Department with digestive hemorrhages, intussusception and even intestinal obstruction.

**Figure 1:**
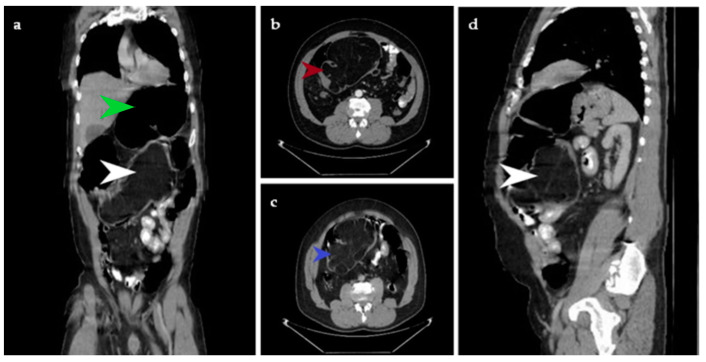
The CT examination with contrast substance reveals, in the coronal plane, voluminous lipoma with measurements of 16/11/12 cm projected in the lumen of the ascending and transverse colon without being able to establish the parietal affiliation. The tumor is indicated with a white arrow. The green arrow shows colonic dilation (**a**). A transverse section in arterial time reveals a voluminous lipoma, indicated with the red arrow (**b**). A cross section in venous time shows a lipomatous tumor, indicated with the blue arrow (**c**). In the sagittal plane, the same tumor is indicated with a white arrow (**d**). We present the case of a 46-year-old patient who arrives to the Brașov Emergency Department for rectal bleeding with fresh blood and blood clots which started about 12 h prior. The arterial blood pressure value was 100/50 mmHg, the heart rate was 100 bpm, and oxygen saturation (SpO_2_) = 96%. The patient had no medical family history. He was known to suffer from stage 2 hypertension, and he was a smoker and occasional alcohol user. All these symptoms appeared against a background of abdominal pain that debuted about 2 years ago, accompanied by intestinal transit disorders: constipation followed by diarrhea that relieves the pain. It was initially decided to hospitalize the patient at the Gastroenterology Clinic for investigations and specialized treatment. General clinical examination revealed pale and sweaty facies, and pale skin and mucous membranes. On examination, the abdomen was distended, mobile with respiratory movements, painful in the right iliac fossa, right flank and epigastrium and with no signs of peritoneal irritation. Rectal examination highlighted a normal-looking perianal region, normotonic anal sphincter, normal-sized prostate with preserved median groove and rectal ampulla that contained fresh blood and blood clots. The paraclinical examination detected Hemoglobin (Hb) 7.7 g/dL, Hematocrit (Ht) 21.6%, Serum iron test 34 ug/dL (normally 37–158 ug/dL). Colonoscopy was performed, and blood traces were detected in the colon and at 100 cm from the anal verge there was a large vegetative formation that occupied the entire lumen and could not be passed with the endoscope.

Lipomas are benign tumors which develop from adipose tissue. The intestinal localization of lipomas is rare, being found in a percentage of 0.2–4.4% of all digestive tumors [1]. Intestinal lipomas were first described by Baurer in 1757 [2]. In terms of their origin, about 90% come from the submucosa and 10% from the subserosa [3]. Although theoretically they can be found anywhere along the digestive tract, from the hypopharynx to the rectum, these tumors are more frequent in the large intestine, rarer in the small intestine and very rare in the esophagus and stomach. The most common clinical manifestations are intussusception, followed by gastrointestinal bleeding when the lining mucosa is ulcerated and intestinal obstruction in the case of large lipomas [4,5,6]. The symptoms are not specific and often these are not taken into account when a patient arrives in the Emergency Department and when being subsequently diagnosed endoscopically or by medical imaging; they are also discovered during surgery.

The paraclinical diagnosis is most often made by performing a colonoscopy that reveals a pediculated tumor formation due to intestinal peristalsis that causes the extrusion of the lipoma in the digestive tract, but can also be sessile. “Pillow sign” occurs when a surface indentation is observed upon pushing the lipoma with closed biopsy forceps during colonoscopy. “Naked fat sign” is the evacuation of fat at the biopsy site [7]. In this case, the colonoscopy examination suggested an ulcerated transverse colon tumor that cannot be passed with the endoscope and the correct diagnosis was revealed by the abdominal CT examination that detected a well-defined tumor with fatty densities of 40–120 HU and allowed the exact establishment of tumor size. Mention should also be made of ‘The squeeze sign’, which is caused by changes in the shape and size of the tumor as a result of intestinal peristalsis and is evident on contrast enhanced fluoroscopy [8]. This examination was not performed in this case. Also, the MRI examination provides the most information for preoperative assessment of the benign or malignant character of lipomatic tumors. Thus, the authors have succeeded in the correct preoperative classification of lipomatic tumors in more than 70% of cases [9].

Small intestinal lipomas, less than 2 cm in diameter, may benefit from endoscopic submucosal excision; for the other cases, limited or extended colon resection surgery is preferable when a malignant neoplastic pathology cannot be excluded [3].

In this case, surgical treatment was the only therapeutic alternative given the giant size of the lipomatous tumor. The laparoscopic approach, although desirable, was not possible due to the impossibility of manipulating the right colon occupied by the large tumor and because of the colonic hemorrhage and worry about an isolated large bowel obstruction. After mechanical preparation of the colon with Fortrans and the administration of prophylactic antibiotic therapy with generation II cephalosporins and Metronidazole, surgery was performed. Extended right hemicolectomy was performed with the ligation at the origin of the ileocolic vessels, right colic and middle colic artery, and the restoration of intestinal continuity was achieved by a latero-lateral ileocolic anastomosis.

**Figure 2:**
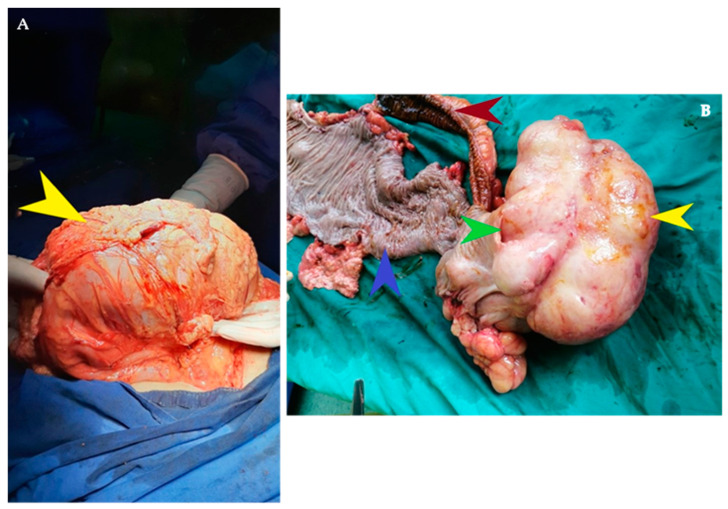
The intraoperative aspect shows a very dilated ascending and transverse colon, with a diameter of 12 cm, occupied by a giant tumor formation which does not invade the intestinal wall of the right colon. The tumor is indicated by the yellow arrow (**A**). The surgical resection specimen is sectioned and a polylobate, lipomatous voluminous tumor formation of 16/12 cm with apparent origin at the level of the ileo-cecal valve was discovered. The yellow arrow indicates the giant lipoma. The ileo-cecal valve is indicated by the green arrow. The ileum is indicated by the red arrow and the ascending colon is shown by the blue arrow (**B**). Usually, colon resections are limited in the case of lipomas because they are benign tumors. In this case, an extended right hemicolectomy was performed in order to be able to safely excise the entire tumor and due to the suspicion of liposarcoma. The postoperative evolution of the patient was favorable, with normal resumption of bowel activity on the 3rd postoperative day and his discharge on the 5th postoperative day.

**Figure 3:**
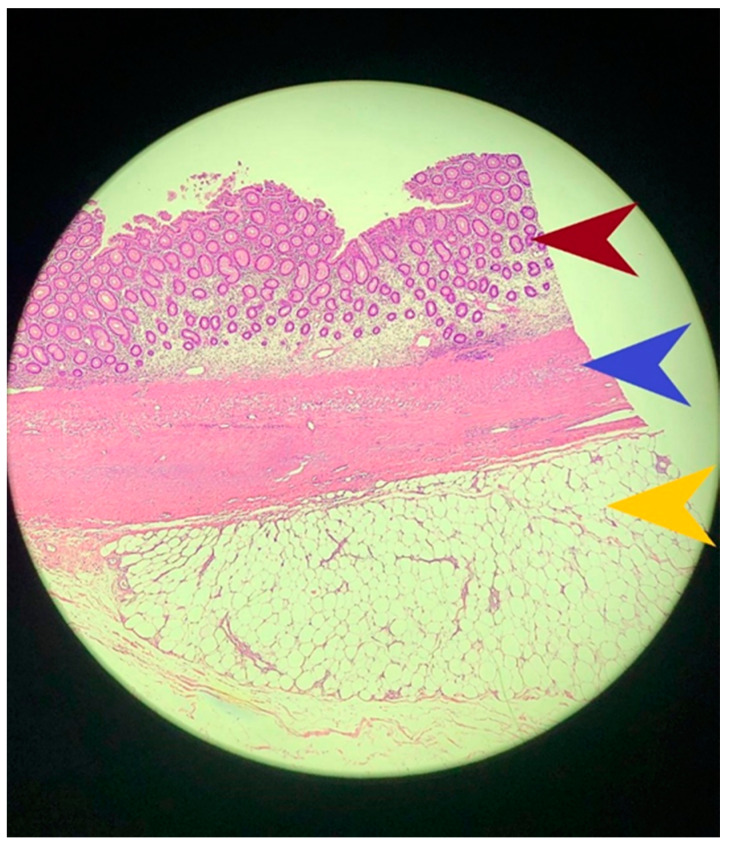
Microscopic image of the lipomatous tumor formation. The red arrow indicates the colic mucosa, the blue arrow shows the muscle of the mucous membrane, and the yellow arrow points to the lipoma originating in the submucous colic. The tumor consists of fat cells without architectural modifications with a mitotic activity of 1.5 mitoses/HPF. The presence of mitotic activity greater than 4.5 mitoses/HPF has a sensitivity of 82.4% and a specificity of 100% for the diagnosis of liposarcoma [10]. The peculiarity of the case is represented by the relatively young age of the patient as well as by the giant size of the tumor. At 3 years post-surgery, the patient remains asymptomatic without any clinical evidence of recurrence. Lipomas are benign tumors rarely found in the colon, accounting for 4% of all benign intestinal tumors [1]. However, these should not be forgotten in patients with the above-mentioned symptoms as they can cause severe complications such as intussusception, intestinal obstruction or gastrointestinal bleeding. It is necessary to establish an appropriate therapeutic conduct and treat these tumors endoscopically or surgically depending on the size.

## Data Availability

The data presented in this study are available on request from the corresponding author.
